# Bioinformatics analysis of prognostic significance of COL10A1 in breast cancer

**DOI:** 10.1042/BSR20193286

**Published:** 2020-02-18

**Authors:** Mingdi Zhang, Hongliang Chen, Maoli Wang, Fang Bai, Kejin Wu

**Affiliations:** Department of Breast Surgery, Obstetrics and Gynecology Hospital of Fudan University, Shanghai, China

**Keywords:** Bioinformatics analysis, breast cancers, COL10A1, Prognosis

## Abstract

Background: Collagen type X alpha 1 (COL10A1) is overexpressed in diverse tumors and displays vital roles in tumorigenesis. However, the prognostic value of COL10A1 in breast cancer remains unclear.

Methods: The expression of COL10A1 was analyzed by the Oncomine database and UALCAN cancer database. The relationship between COL10A1 expression level and clinical indicators including prognostic data in breast cancer were analyzed by the Kaplan–Meier Plotter, PrognoScan, and Breast Cancer Gene-Expression Miner (bc-GenExMiner) databases.

Results: COL10A1 was up-regulated in different subtypes of breast cancer. Estrogen receptor (ER), progesterone receptor (PR), human epidermal growth factor receptor-2 (HER-2) status and nodal status were positively correlated with COL10A1 expression. Conversely, age, the Scarff–Bloom–Richardson (SBR) grade, basal-like status, and triple-negative status were negatively related to COL10A1 level in breast cancer samples compared with normal tissues. Patients with increased COL10A1 expression level showed worse overall survival (OS), relapse-free survival (RFS), distant metastasis-free survival (DMFS) and disease-free survival (DFS). COL10A1 was positively correlated with metastatic relapse-free survival. GSEA analysis revealed that enrichment of TGF-β signaling pathway. 15-leucine-rich repeat containing membrane protein (*LRRC15*) is a correlated gene of COL10A1.

Conclusion: Bioinformatics analysis revealed that COL10A1 might be considered as a predictive biomarker for prognosis of breast cancer. Further experiments and clinical trials are essential to elucidate the value of COL10A1 in breast cancer treatment.

## Introduction

Breast cancer is the most common cancer among women and a main cause of cancer-related deaths worldwide [1]. Screening and diagnosis of early breast cancer are crucial to reduce morbidity and mortality [2,3]. Although clinical, pathological and molecular indicators are generally applicated in predicting prognosis, the underlying pathogenesis of breast cancer aggressiveness remain poorly understood, and minimally invasive biomarkers for the detection of early-stage breast cancer are vital in breast cancer research [4].

Collagen type X alpha 1 (*COL10A1*), a secreted, short-chain collagen, belongs to the collagen family, which is a major interstitial matrix component [5]. COL10A1 expression is elevated in many solid tumor types, such as colon cancer, esophagus cancer, and breast cancer, and displays vital roles in many critical cellular processes such as cell proliferation, migration, invasion and tumor vasculature [6–8]. COL10A1 protein levels in plasma might be a potential diagnostic predictor for early breast cancer [9]. Although COL10A1 was reported to be highly expressed in tumors by high throughput sequencing, the specific role of COL10A1 in breast cancer was unknown [10–12].

Therefore, in the present study, we evaluated the significance of COL10A1 gene expression in breast cancer by using comprehensive bioinformatics analysis of the clinical indicators and survival data in several large online databases.

## Materials and methods

### Oncomine analysis

The COL10A1 mRNA expression level was analyzed in breast cancer and matched normal tissues based on the Oncomine Platform (www.oncomine.org). The analysis was conducted using the following filters: Gene, COL10A1; differential analysis, cancer vs. normal analysis; cancer type, breast cancer; and data type, mRNA. In the present study, we selected two-fold change, *P*-value = 1E-4 and top 10% gene rank as threshold. All statistical methods and statistical values were obtained directly from the corresponding database.

### Breast cancer gene-expression miner

The expression of COL10A1 mRNA in different subtypes of breast cancer and the correlation between genes or identified clusters of correlated co-expressed genes were analyzed using the Breast Cancer Gene-Expression Miner (bcGenExMiner v4.3, http://bcgenex.centregauducheau.fr/BC-GEM). The correlation between COL10A1 and 15-leucine-rich repeat containing membrane protein (LRRC15) were generated using the correlation module.

### UALCAN cancer database

UALCAN is a comprehensive, user-friendly and interactive web resource for analyzing cancer OMICS data. It is built on PERL-CGI with high quality graphics using javascript and CSS. UALCAN now provides protein expression analysis option using data from Clinical Proteomic Tumor Analysis Consortium (CPTAC) Confirmatory/Discovery dataset. We evaluated the protein expression of COL10A1 in breast cancer by CPTAC analysis.

### PrognoScan

The PrognoScan online database (http://www.prognoscan.org/) provides a powerful platform for assessing the biological relationships between gene expression and prognostic information in cancer patients. PrognoScan includes public microarray datasets with clinical annotation of gene expression and prognosis from Gene Expression Omnibus (GEO), ArrayExpress and individual laboratory websites. The correlation between COL10A1 expression and survival in breast cancers was analyzed by PrognoScan database. Cox *P*-values and hazard ratio (HR) with 95% confidence intervals were calculated automatically according to the mRNA level (high or low).

### Kaplan–Meier survival curve analysis

The prognostic value of COL10A1 mRNA and protein expression in breast cancer was assessed according to overall survival (OS)/relapse-free survival (RFS) using Kaplan–Meier plotter (kmplot.com/analysis), an online database including gene expression data and clinical data. With the purpose to assess prognostic value of a specific gene, the patient samples were divided into two cohorts according to the median expression of the gene (high vs. low expression). Log-rank *P*-values and HRs with 95% confidence intervals were determined on the webpage.

### UCSC Xena

The heat map of COL10A1 and LRRC15 in the same patient cohort were constructed by data mining in the Cancer Genome Atlas (TCGA) Breast Cancer using the UCSC Xena browser (http://xena.ucsc.edu/).

### Gene Expression Profiling Interactive Analysis dataset

Gene Expression Profiling Interactive Analysis (GEPIA) is a newly developed interactive web server for analyzing the RNA sequencing expression data of 9736 tumors and 8587 normal samples from TCGA and the Genotype-Tissue Expression (GTEx) projects, using a standard processing pipeline (http://gepia.cancer-pku.cn/). GEPIA provides customizable functions such as tumor or normal differential expression analysis, profiling according to cancer types or pathological stages, patient survival analysis, similar gene detection, correlation analysis and dimensionality reduction analysis. The expression of LRRC15 was analyzed by GEPIA database.

### LinkedOmics dataset

LinkedOmics (http://www.linkedomics.orglogin.php) is a new and unique tool in the software ecosystem for disseminating data from large-scale cancer omics projects. It uses preprocessed and normalized data from the Broad TCGA Firehose and Clinical Proteomic Tumor Analysis (CPTAC) data portal to reduce redundant efforts and focus on the discovery and interpretation of attribute associations, and thus complements existing cancer data portals. GSEA analysis was conducted by LinkedOmics Dataset.

## Results

### The expression of COL10A1 is increased in breast cancer patients

**Figure 1:**
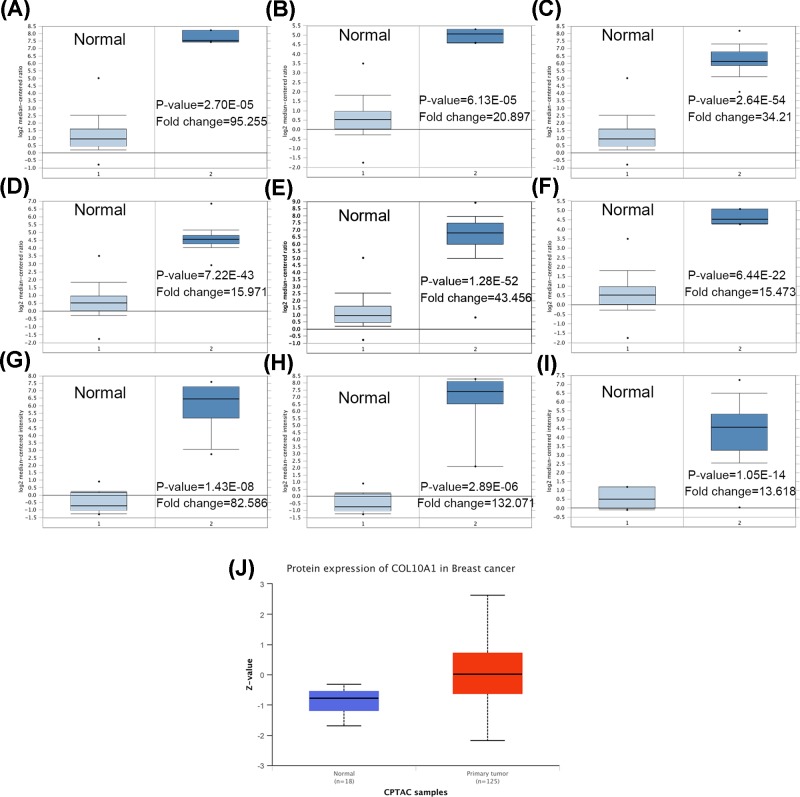
The mRNA and protein expression of COL10A1 in breast cancer compared to normal The mRNA expression of COL10A1 in different subtypes of breast cancer compared to normal individuals derived from the Oncomine database. Data shown for male breast carcinoma (**A**), intraductal cribriform breast adenocarcinoma (**B**), invasive breast carcinoma (**C**), invasive lobular breast carcinoma (**D**), invasive ductal breast carcinoma (**E**), mixed lobular and ductal breast carcinoma (**F**), ductal breast carcinoma *in situ* stroma (**G**), invasive ductal breast carcinoma stroma (**H**) and ductal breast carcinoma (**I**). * stands for the maximum and minimum values. (**J**) The protein expression of COL10A1 in primary breast cancer and normal tissues analyzed by UALCAN cancer database. Z-values represent standard deviations from the median across samples for the given cancer type. Log2 Spectral count ratio values from CPTAC were first normalized within each sample profile, then normalized across samples.

The mRNA expression of COL10A1 in breast cancer was analyzed using the Oncomine database. The higher expression of COL10A1 was observed in male breast carcinoma, intraductal cribriform breast adenocarcinoma, invasive breast carcinoma, invasive lobular breast carcinoma, invasive ductal breast carcinoma, mixed lobular and ductal breast carcinoma, ductal breast carcinoma *in situ* stroma, invasive ductal breast carcinoma stroma and ductal breast carcinoma, compared with the corresponding normal tissues (Figure 1A-I and Table 1). The higher protein expression of COL10A1 was also detected in breast cancer tissues by UALCAN cancer database (Figure 1J).

**Table 1:** COL10A1 expression in different subtypes of breast cancer and normal tissues using the Oncomine database

Breast cancer subtype	*P*-value	*t* test	Fold change	Patient number	Reference
Male breast carcinoma	2.70E-05	23.123	95.255	3	TCGA
Intraductal cribriform breast adenocarcinoma	6.13E-05	17.962	20.897	3	TCGA
Invasive breast carcinoma	2.64E-54	29.787	34.21	76	TCGA
Invasive lobular breast carcinoma	7.22E-43	24.558	15.971	36	TCGA
Invasive ductal breast carcinoma	1.28E-52	35.009	43.456	389	TCGA
Mixed lobular and ductal breast carcinoma	6.44E-22	24.786	15.473	7	TCGA
Ductal breast carcinoma *in situ* stroma	1.43E-08	12.048	82.586	11	PMID: 19187537
Invasive ductal breast carcinoma stroma	2.89E-06	9.457	132.071	9	PMID: 19187537
Ductal breast carcinoma	1.05E-14	12.111	13.618	40	PMID: 16473279

### The relationship between COL10A1 expression and clinical indicators in breast cancer patients

**Figure 2:**
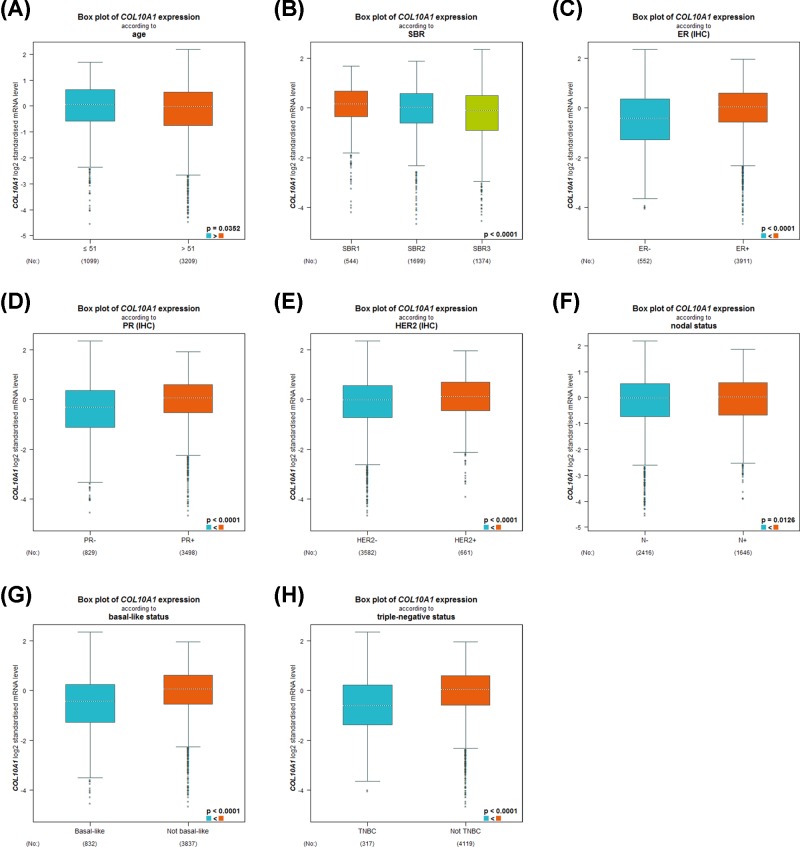
Box plot revealing the relationship between COL10A1 expression and different clinical indicators using the bc-GenExMiner software Data shown for age (**A**), SBR (**B**), ER (**C**), PR (**D**), HER-2 (**E**), nodal status (**F**), basal-like status (**G**) and triple-negative status (**H**).

By using the bc-GenExMiner online tool, we next compared COL10A1 expression among groups of patients, according to different clinical indicators. Regarding age, the expression of COL10A1 was significantly higher in ≤51- compared with >51-year group (Figure 2A and Table 2). The Scarff–Bloom–Richardson (SBR) is a histological grade that evaluates tubule formation, nuclear characteristics of pleiomorphism and mitotic index. Breast cancer patients with more advanced SBR grade tended to express lower *COL10A1* gene (Figure 2B and Table 2). Estrogen receptor (ER), progesterone receptor (PR) status and human epidermal growth factor receptor-2 (HER-2) status were positively associated with COL10A1 expression (Figure 2C–E and Table 2). Breast cancer patients with positive nodal status (N) showed increased level of COL10A1 than those with negative nodal status (Figure 2F and Table 2). Besides, we found that COL10A1 was strongly elevated in non-basal-like subtype with respect to basal-like subtype; the same pattern of change was also observed in triple-negative breast cancer (TNBC) patients (Figure 2G,H and Table 2).

**Table 2:** Relationship between COL10A1 expression and clinical parameters of breast cancer patients using the bc-GenExMiner database

Variables	Patient number	COL10A1 mRNA	*P*-value
Age (years)			0.0352
≤51	1099	Increased	
>51	3209		
**ER**			<0.0001
Negative	552		
Positive	3911	Increased	
**PR**			<0.0001
Negative	829		
Positive	3498	Increased	
**HER-2**			<0.0001
Negative	3582		
Positive	661	Increased	
**Nodal status**			0.0126
Negative	2416		
Positive	1646	Increased	
**Basal-like status**			<0.0001
Non-basal-like	832		
basal-like	3837	Increased	
**Triple-negative status**			<0.0001
Non-triple-negative	317		
Triple-negative	4119	Increased	

### Increased expression of COL10A1 correlates with poor outcome in breast cancer patients

**Figure 3:**
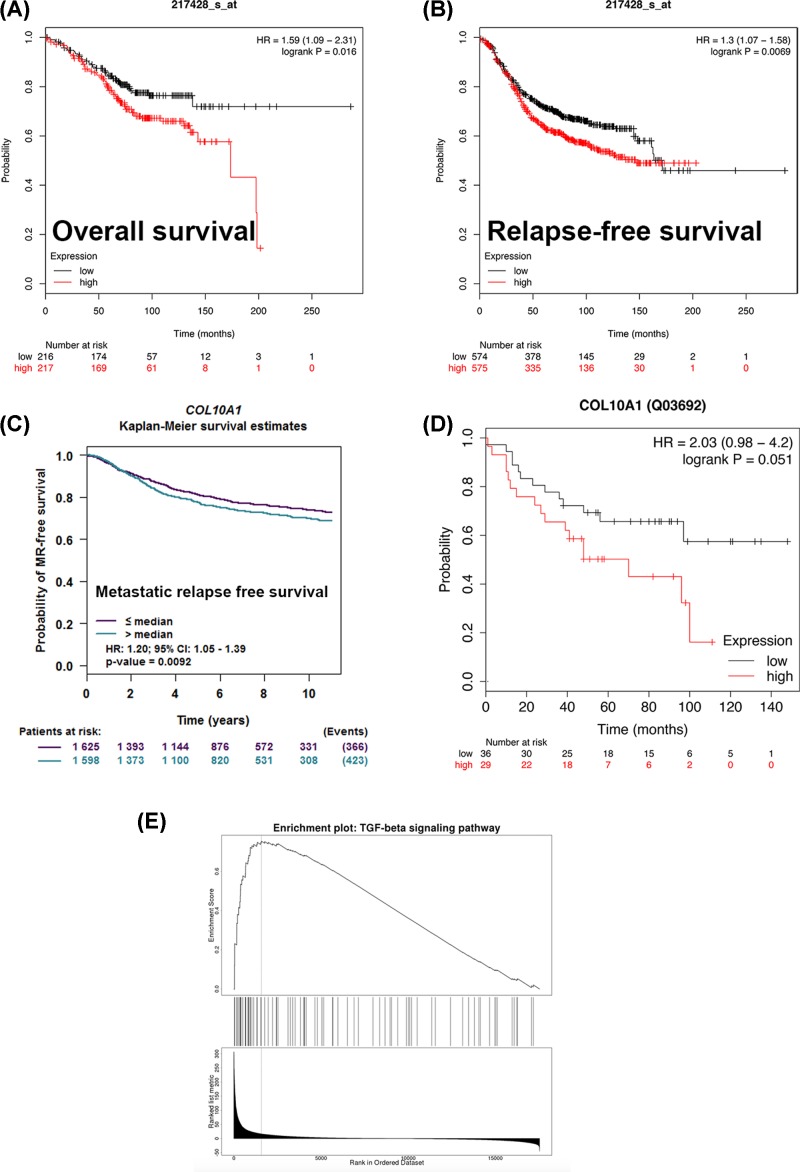
Survival data evaluating the prognostic value of COL10A1 and the GSEA analysis Analysis is shown for OS (**A**), RFS (**B**) by Kaplan–Meier Plotter and metastatic RFS (**C**) by bc-GenExMiner database. (**D**) The relationship between the protein expression of COL10A1 and prognosis by Kaplan–Meier Plotter (**E**) GSEA analysis obtained from LinkedOmics Dataset.

We then analyzed the prognostic value of *COL10A1* gene. The Kaplan–Meier plotter revealed that lower level of COL10A1 correlated with preferable OS (Figure 3A). While breast cancer patients with up-regulated COL10A1 demonstrated worse RFS (Figure 3B). Furthermore, the PrognoScan database showed that overexpression of COL10A1 was significantly associated with inferior OS, relapse-free survival, distant metastasis-free survival and disease-free survival (Table 3). To further investigate the role of COL10A1 in breast cancer prognosis, we verified that COL10A1 was positively correlated with metastatic RFS by the bc-GenExMiner software (Figure 3C). We also detected the high regulation of COL10A1 protein was mild significantly related to the worse OS in Kaplan–Meier plotter (Figure 3D). To identify the potential function of COL10A1, GSEA was conducted to search KEGG pathways enriched. The results revealed that enrichment of TGF-β signaling pathway (Figure 3E).

**Table 3:** COL10A1 expression and survival data of breast cancer patients using the PrognoScan database

Dataset	End point	Probe ID	*n*	Minimum *P*-value	HR
GSE2034	Distant metastasis-free survival	217428_s_at	286	0.000232858	1.30 [1.06–1.58]
GSE1379	RFS	17001	60	0.00644422	1.76 [1.03–3.01]
GSE7849	Disease-free survival	38566_at	76	0.0097891	1.82 [0.89–3.70]
GSE7390	OS	217428_s_at	198	0.0260043	1.12 [0.96–1.31]
GSE7390	RFS	217428_s_at	198	0.030156	1.06 [0.95–1.19]
GSE7390	Distant metastasis-free survival	217428_s_at	198	0.0325797	1.13 [0.97–1.31]

### COL10A1 and LRRC15 are co-expressed in breast cancer patients

**Figure 4:**
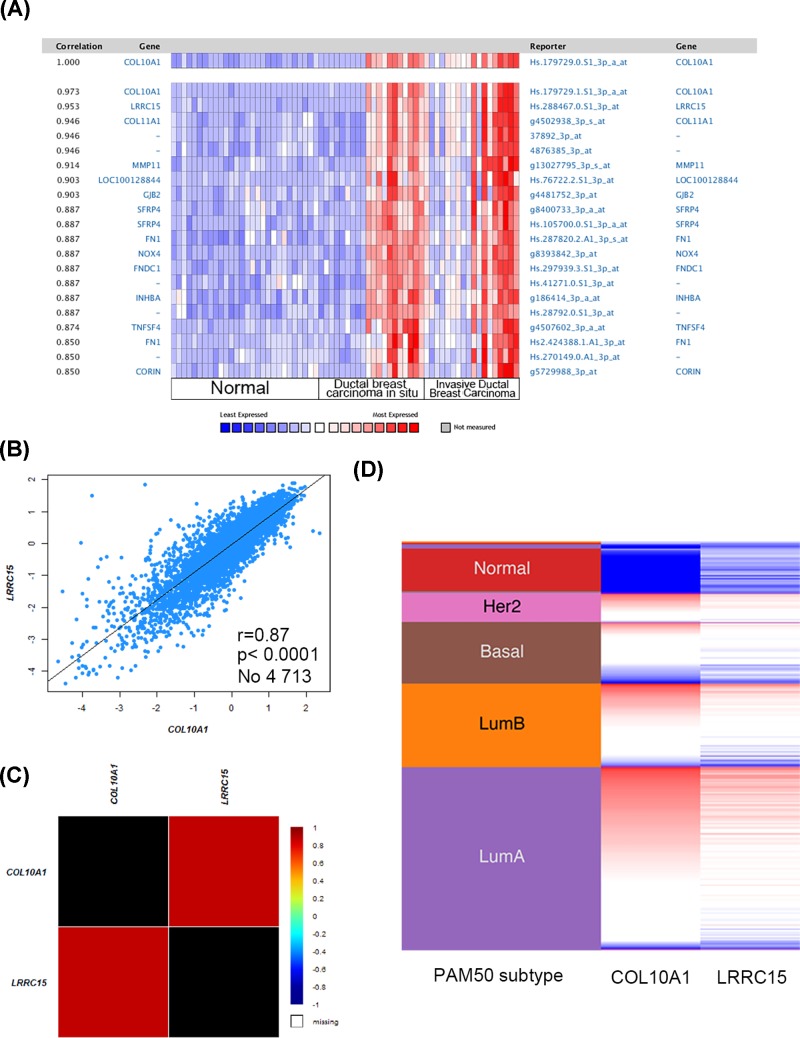
Co-expression analysis of COL10A1 (**A**) Co-expression profile of COL10A1 identified using the Oncomine database. (**B,C**) Showed the correlation between COL10A1 and LRRC15 expression in breast cancer by the bc-GenExMiner software. (**D**) Heat map of COL10A1 and LRRC15 expression across PAM50 breast cancer subtypes in the TCGA database obtained from the UCSC Xena web-based tool.

To further study the underlying mechanism of COL10A1 in breast cancer, we conducted co-expression data mining of COL10A1 by the Oncomine database. The co-expression profile of COL10A1 was identified with a large cluster of 19139 genes across 66 breast carcinomas, and LRRC15 is a correlated gene (Figure 4A). Further analysis using bc-GenExMiner revealed the correlation between COL10A1 and LRRC15 (Figure 4B,C). By comparing the COL10A1 and LRRC15 expression heat map derived from the UCSC Xena web-based tool (Figure 4D), COL10A1 expression was proved to be positively related with LRRC15 transcript level, which was determined among a 50-gene qPCR assay (PAM50) breast cancer subtypes in TCGA database (Figure 4C). The data above indicated that COL10A1 could be associated with the LRRC15 signaling pathways in breast cancer.

### The validation and survival analysis of LRRC15 in breast cancer

**Figure 5:**
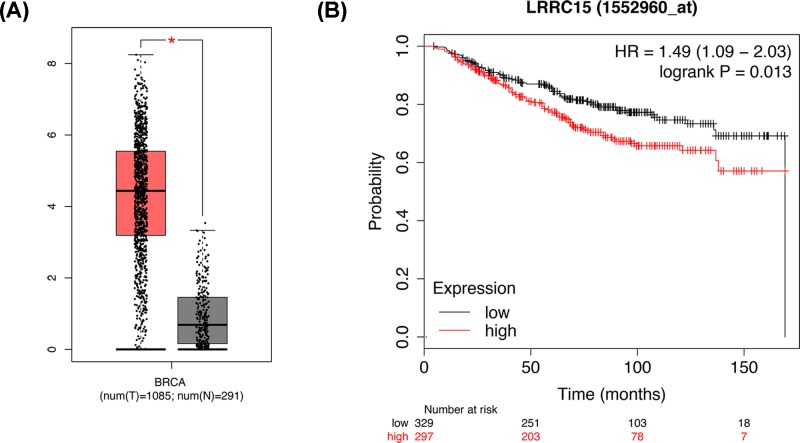
The expression and survival data indicating the potential function of LRRC15 in breast cancer (**A**) The expression of LRRC15 showed by GEPIA database. (**B**) The OS status for the expression of LRRC15 from Kaplan–Meier Plotter.

The expression of LRRC15 were validated in GEPIA database. We unearthed that LRRC15 were significantly up-regulated in breast cancer tissues (Figure 5A). The survival analysis in Kaplan–Meier plotter database confirmed that the up-regulation of LRRC15 were correlated with shorter OS of breast cancer patients, respectively (Figure 5B).

## Discussion

Breast cancer has been reported to be associated with the aberrant expression of oncogenes [13]. Despite the improvements in the diagnosis, treatment and prognosis prediction of breast cancer, it remains the most prevalent malignant tumor with the high incidence in women worldwide. The identification of novel biomarkers for breast cancer is crucial to its diagnosis, therapy and prognosis [14].

COL10A1 is a member of the collagen family. COL10A1 encodes the α chain of type X collagen, a short chain collagen expressed by hypertrophic chondrocytes during endochondral ossification. The expression of COL10A1 was increased in various solid human tumor tissues, which contributed to tumor vasculature staining [6]. COL10A1 showed an important role in differentiating *in situ* from invasive breast cancer and characterizing DCIS with a high risk developing IDC [11,15,16]. Additionally, the concentration of COL10A1 in the plasma could be a potential biomarker to discriminate breast cancer patients from those with benign disease [9]. Of interest, increased expression of COL10A1 correlate with poor pathologic response in breast tumors [17]. However, the significance of COL10A1 expression in the prognosis of breast cancer remains unclear. This is the first study to identify COL10A1 as a potential predictive biomarker for prognosis of breast cancer.

In our study, we analyzed the expression profile of COL10A1 by Oncomine database. COL10A1 was higher expressed in male breast carcinoma, intraductal cribriform breast adenocarcinoma, invasive breast carcinoma, invasive lobular breast carcinoma, invasive ductal breast carcinoma, mixed Lobular and Ductal Breast Carcinoma, ductal breast carcinoma *in situ* stroma, invasive ductal breast carcinoma stroma and ductal breast carcinoma with respect to normal tissues. The bc-GenExMiner online tool revealed that ER, PR, HER-2 status and nodal status were positively correlated with COL10A1 expression. Conversely, age, SBR, basal-like status and triple-negative status were negatively related to COL10A1 level in breast cancer samples compared with normal tissues. Therefore, these results indicated that the expression of COL10A1 may predict the prognosis of breast cancer.

We further investigated the prognostic value of COL10A1 in breast cancer using the Kaplan–Meier Plotter, PrognoScan and bc-GenExMiner databases. Patients with increased COL10A1 showed worse OS, RFS, distant metastasis-free survival and disease-free survival. These findings collectively elucidated that the expression of COL10A1 might be a predictive biomarker for prognosis of breast cancer.

GSEA analysis revealed the function of COL10A1 enriched in TGF-β signaling pathway in breast cancer. The previous study elucidated the mechanistic link between COL10A1 and the TGF-β1–SOX9 axis in gastric cancer progression [8]. Our study is the first to reveal the potential function between COL10A1 and TGF-β signaling pathway in breast cancer.

The co-expression of COL10A1 was analyzed using the Oncomine, bc-GenExMiner and UCSC Xena web-based tools. The expression of LRRC15 was positively correlated with COL10A1 expression. The type I transmembrane protein LRRC15 is a member of the LRR superfamily [18,19]. The LRR family is a structural module for protein–protein and protein–matrix interactions used for molecular recognition process such as cell adhesion, signal transduction, DNA repair and RNA processing [20,21]. LRRC15 was found to be highly expressed on the cell surface of stromal fibroblasts in many solid tumors. Additionally, LRRC15 was considered as a new marker of cancer-associated fibroblasts and cancers of mesenchymal origin and might be applicated in antibody–drug conjugate targeting the tumor stroma [22].

In conclusion, this analysis revealed that COL10A1 was higher expressed in breast cancer compared with normal tissues and was correlated with a worse survival. COL10A1 could be considered as a predictive biomarker for prognosis of breast cancer with co-expressed LRRC15. Further experiments and clinical trials are essential to elucidate the value of COL10A1 in breast cancer treatment.

## Abbreviations

CGI: Common Gateway Interface
COL10A1: Collagen type X alpha 1
CPTAC: Clinical Proteomic Tumor Analysis Consortium
CSS: Cascading Style Sheets
DCIS: Ductal carcinoma in situ
ER: Estrogen receptor
GEPIA: Gene Expression Profiling Interactive Analysis
GSEA: Gene Set Enrichment Analysis
HER-2: Human epidermal growth factor receptor-2
HR: Hazard ratio
IDC: Intraductal carcinoma of the breast
KEGG: Kyoto Encyclopedia of Genes and Genomes
LRRC15: 15-leucine-rich repeat containing membrane protein
OS: Overall survival
PR: Progesterone receptor
RFS: Relapse-free survival
SBR: Scarff–Bloom–Richardson
TCGA: The Cancer Genome Atlas

## References

[1] Najafi S., Sadeghi M., Abasvandi F., Shajari M.R., Mohebi K. and Ghandchi H. (2019) Prognostic factors influencing prognosis in early breast cancer patients. Prz. Menopauzalny 18, 82–88 31485204 10.5114/pm.2019.86833 PMC6719640

[2] Mayor S. (2015) Screening for early breast cancer reduces invasive cancer, study finds. BMJ 351, h6576 10.1136/bmj.h6576 26643040

[3] Hamann M., Grill S., Struck J., Bergmann A., Hartmann O., Polcher M. et al. (2019) Detection of early breast cancer beyond mammographic screening: a promising biomarker panel. Biomark. Med. 10.2217/bmm-2019-0085 31468986

[4] Arnedos M., Roulleaux Degage M., Perez-Garcia J. and Cortes J. (2019) Window of Opportunity trials for biomarker discovery in breast cancer. Curr. Opin. Oncol. 31, 486–492 31464762 10.1097/CCO.0000000000000583

[5] Zhang X., Liang H., Liu W., Li X., Zhang W. and Shang X. (2019) A novel sequence variant in COL10A1 causing spondylometaphyseal dysplasia accompanied with coxa valga: a case report. Medicine (Baltimore) 98, e16485 10.1097/MD.0000000000016485 31348255 PMC6708723

[6] Chapman K.B., Prendes M.J., Sternberg H., Kidd J.L., Funk W.D., Wagner J. et al. (2012) COL10A1 expression is elevated in diverse solid tumor types and is associated with tumor vasculature. Future Oncol. 8, 1031–1040 10.2217/fon.12.79 22894674

[7] Huang H., Li T., Ye G., Zhao L., Zhang Z., Mo D. et al. (2018) High expression of COL10A1 is associated with poor prognosis in colorectal cancer. Onco Targets Ther. 11, 1571–1581 10.2147/OTT.S160196 29593423 PMC5865565

[8] Li T., Huang H., Shi G., Zhao L., Li T., Zhang Z. et al. (2018) TGF-beta1-SOX9 axis-inducible COL10A1 promotes invasion and metastasis in gastric cancer via epithelial-to-mesenchymal transition. Cell Death Dis. 9, 849 10.1038/s41419-018-0877-2 30154451 PMC6113209

[9] Giussani M., Landoni E., Merlino G., Turdo F., Veneroni S., Paolini B. et al. (2018) Extracellular matrix proteins as diagnostic markers of breast carcinoma. J. Cell. Physiol. 233, 6280–6290 10.1002/jcp.26513 29521413

[10] Malvia S., Bagadi S.A.R., Pradhan D., Chintamani C., Bhatnagar A., Arora D. et al. (2019) Study of gene expression profiles of breast cancers in Indian women. Sci. Rep. 9, 10018 10.1038/s41598-019-46261-1 31292488 PMC6620270

[11] Chang H.J., Yang M.J., Yang Y.H., Hou M.F., Hsueh E.J. and Lin S.R. (2009) MMP13 is potentially a new tumor marker for breast cancer diagnosis. Oncol. Rep. 22, 1119–1127 19787229 10.3892/or_00000544

[12] Makoukji J., Makhoul N.J., Khalil M., El-Sitt S., Aldin E.S., Jabbour M. et al. (2016) Gene expression profiling of breast cancer in Lebanese women. Sci. Rep. 6, 36639 10.1038/srep36639 27857161 PMC5114572

[13] Zu C., Zhang M., Xue H., Cai X., Zhao L., He A. et al. (2015) Emodin induces apoptosis of human breast cancer cells by modulating the expression of apoptosis-related genes. Oncol. Lett. 10, 2919–2924 10.3892/ol.2015.3646 26722264 PMC4665964

[14] Zu C., Qin G., Yang C., Liu N., He A., Zhang M. et al. (2018) Low dose Emodin induces tumor senescence for boosting breast cancer chemotherapy via silencing NRARP. Biochem. Biophys. Res. Commun. 505, 973–978 10.1016/j.bbrc.2018.09.045 30274778

[15] Desmedt C., Majjaj S., Kheddoumi N., Singhal S.K., Haibe-Kains B., El Ouriaghli F. et al. (2012) Characterization and clinical evaluation of CD10+ stroma cells in the breast cancer microenvironment. Clin. Cancer Res. 18, 1004–1014 10.1158/1078-0432.CCR-11-0383 22235100 PMC4446057

[16] Schultz S., Bartsch H., Sotlar K., Petat-Dutter K., Bonin M., Kahlert S. et al. (2018) Progression-specific genes identified in microdissected formalin-fixed and paraffin-embedded tissue containing matched ductal carcinoma in situ and invasive ductal breast cancers. BMC Med. Genomics 11, 80 10.1186/s12920-018-0403-5 30236106 PMC6147035

[17] Brodsky A.S., Xiong J., Yang D., Schorl C., Fenton M.A., Graves T.A. et al. (2016) Identification of stromal ColXalpha1 and tumor-infiltrating lymphocytes as putative predictive markers of neoadjuvant therapy in estrogen receptor-positive/HER2-positive breast cancer. BMC Cancer 16, 274 10.1186/s12885-016-2302-5 27090210 PMC4835834

[18] Wang Y., Liu Y., Zhang M., Lv L., Zhang X., Zhang P. et al. (2018) LRRC15 promotes osteogenic differentiation of mesenchymal stem cells by modulating p65 cytoplasmic/nuclear translocation. Stem Cell Res. Ther. 9, 65 10.1186/s13287-018-0809-1 29523191 PMC5845373

[19] O’Prey J., Wilkinson S. and Ryan K.M. (2008) Tumor antigen LRRC15 impedes adenoviral infection: implications for virus-based cancer therapy. J. Virol. 82, 5933–5939 10.1128/JVI.02273-07 18385238 PMC2395123

[20] Kobe B. and Deisenhofer J. (1994) The leucine-rich repeat: a versatile binding motif. Trends Biochem. Sci. 19, 415–421 10.1016/0968-0004(94)90090-6 7817399

[21] Kobe B. and Kajava A.V. (2001) The leucine-rich repeat as a protein recognition motif. Curr. Opin. Struct. Biol. 11, 725–732 10.1016/S0959-440X(01)00266-4 11751054

[22] Purcell J.W., Tanlimco S.G., Hickson J., Fox M., Sho M., Durkin L. et al. (2018) LRRC15 is a novel mesenchymal protein and stromal target for antibody-drug conjugates. Cancer Res. 78, 4059–4072 10.1158/0008-5472.CAN-18-0327 29764866

